# Assessing health-seeking behaviour and malaria prevention practices among communities in four districts of the Volta Region of Ghana

**DOI:** 10.1186/s12936-021-03986-7

**Published:** 2021-11-27

**Authors:** Verner N. Orish, Raymond Saa-Eru Maalman, Otchere Y. Donkor, Barbara Yordanis Henandez Ceruantes, Eric Osei, Hubert Amu, Prince Kubi Appiah, Kennedy Diema Konlan, Hadiru Mumuni, Eunji Kim, Siwoo Kim, Hajun Jung, Jones Ofori-Amoah, Philip Kofie, Martin Adjuik, Robert Kaba Alhassan, Ernestina Safoa Donkor, Francis Bruno Zottor, Margaret Kweku, Paul Amuna, So Yoo Kim, John Owusu Gyapong, the UHAS-Yonsei Project Team

**Affiliations:** 1 School of Medicine, University of Health and Allied Sciences, Ho, Ghana; 2 School of Public Health, University of Health and Allied Sciences, Hohoe, Ghana; 3 Department of Public Health Graduate School, Yonsei University, Seoul, Republic of Korea; 4 School of Nursing and Midwifery, University of Health and Allied Sciences, Ho, Ghana; 5 Korea Foundation for International Healthcare Ghana Office, Accra, Ghana; 6 Asian Institute for Bioethics and Health Law, College of Medicine, Yonsei University, Seoul, Republic of Korea; 7 Directorate of International Affairs, University of Health and Allied Sciences, Ho, Ghana; 8 Office of the Vice-Chancellor, University of Health and Allied Sciences, Ho, Ghana

**Keywords:** Malaria, Insecticide treated nets (ITNs), Malaria prevention, Control, Sub-Saharan Africa, Ghana

## Abstract

**Background:**

Malaria is a preventable disease that causes huge morbidity and mortality in malaria-endemic areas, especially among children and pregnant women. The malaria control programme focuses on the prevention of mosquito bites using insecticide-treated nets (ITNs) and mosquito aerosol sprays and coils, as well as prevention of severe disease among those infected through prompt and adequate treatment. The success of the malaria control programme in Ghana is dependent on the malaria prevention practices of people in the community. Therefore, this study evaluated the malaria prevention practices of participants in four districts of the Volta Region of Ghana.

**Methods:**

This was a cross-sectional study conducted in Ketu South, Nkwanta South, Hohoe Municipality and Ho West districts of the Volta Region of Ghana. Questionnaire were administered to adults who consented to each household visited. Questions were asked on the socio-demographics and malaria prevention practices of the households. Data analysis was done using SPSS version 23 with frequency distribution done for all the variables. Pearson chi-square was used to determine the significant association between socio-demographics and malaria prevention practices, and Multivariate nominal logistic regression analysis was used to model the relationship between dichotomous dependent variables (ITN ownership and usage) and independent variables.

**Results:**

Out of the 2493 participants; 2234 (89.6%) owned ITN and 1528 (68.4%) used ITN a night before this study, 768 (30.8%) used mosquito aerosol spray and 368 (15%) used mosquito coil. More females significantly owned ITN than males (1293, 92.4%, p ≤ 0.001). Participants from Ketu South had 1.5 times higher odds of owning an ITN compared to Ho West whose odds are not different from Nkwanta South or Hohoe (AOR, 1.56 [95% 1.09–2.22]; p = 0.01). In terms of ITN usage, participants in Nkwanta South were less likely to use ITN compared to the other districts; AOR, 0.434 [95% CI 0.31–0.62, p < 0.001**].** Also, of the 668 participants that had a fever within the past 3 days, 268 (40.1%) visited a patent medicine store and 156 (23.4%) visited health facilities.

**Conclusion:**

There is high ownership of ITNs, but relatively low utilization among the community members. Education on malaria prevention practices should be intensified and continuous among the population of the Volta Region to ensure the success of malaria control in the region.

## Background

Malaria is a serious public health issue causing preventable morbidity and mortality in malaria-endemic areas worldwide, especially in sub-Saharan African [1, 2]. In sub-Saharan Africa, where *Plasmodium falciparum* is responsible for the majority of malaria cases, there is an unevenly high burden of malaria, constituting about 93% and 94% of the global 409 million malaria cases and 229 million deaths, respectively [2]**.** These result in an estimated loss of over 35 million Disability Adjusted life years [1–3]. Despite the appreciable reduction of malaria cases and deaths in the region due to control and prevention programmes, malaria still poses a threat to million**s** of lives in sub-Saharan Africa [1, 2].

Malaria control and prevention has evolved over the years, with the World Health Organization (WHO) initiation of the Rollback malaria programme in 1998 focused on reducing malaria cases by 50% by the year 2010. The WHO Global Technical Strategy for Malaria 2016–2030 focuses on the reduction of malaria burden by 90% by the year 2030 [4, 5]. In summary, malaria control and prevention is focused on mortality and infection prevention. Mortality prevention employs the strategy of adequate case management involving prompt diagnosis followed by effective treatment; while infection prevention uses vector control strategies like eradication of mosquitoes through indoor and outdoor sprayings, and preventing human bite using insecticide-treated bed nets (ITNs) [5–7]. One of the major challenges of these control strategies is inadequate prevention practices among the targeted population or communities [6].

Malaria prevention practices among individuals in a community is one of the factors that contribute to the success of malaria control programmes in target populations [7]. How well individuals in a community embrace and assimilate the practices of malaria control and prevention activities will determine the success of the programme in achieving the desired outcomes of reduced malaria morbidity and mortality in the community [6, 7]. Health-seeking behaviour for malaria treatment is fundamental in the success of mortality prevention of malaria [8, 9]. Some decisions taken after the onset of putative malaria symptoms include staying home and doing nothing, treatment with herbal medication, self-medication with over-the-counter drugs, and visits to the health facilities [8, 9]. Fever is the most common symptom for malaria, but unfortunately shares commonality with other febrile illnesses that are also prevalent in malaria endemic areas [10]. It is, therefore, pertinent that persons with fever should seek appropriate care to rule out malaria or other causes of febrile illness [10]. Any decision that does not ultimately result in prompt diagnosis and treatment might result in fatal consequences, especially among children [11]. The ITN is a very beneficial malaria prevention tool [12] that has still not reached 100% uptake in many malaria-endemic areas [12, 13]. Though the mass distribution of ITN has been employed, its uptake has been marred by several logistics challenges; as well as poor usage by households who own ITNs, caused by several factors including inadequate number of ITNs for all the members of the household [13]. Insecticidal indoor spraying, a vector control strategy that is commonly practised in malaria-endemic areas, is also effective in preventing bites from nuisance mosquitoes [14]. It involves insecticides in aerosol spray cans and mosquito coils which target adult flying or resting mosquitoes [15–17].

In Ghana, malaria is still endemic, contributing to 4% and 7% of global malaria cases and malaria burden in West Africa, respectively [18, 19]. Malaria control and prevention in Ghana has undoubtedly yielded some desired outcomes with close to 50% reduction in malaria cases from 2005 to 2015, and a decline in malaria-related deaths from 19% to about 4.2% in 2016 [18, 19]. With the early participation in the Rollback malaria programme, Ghana has made enviable strides in ITN campaign and coverage in the country, with 73% household ownership in 2016 from 68% 2 years earlier [20]. With the availability of effective treatment and an improvement in easy access to healthcare through the national health insurance scheme, in addition to availability of primary healthcare centres in the communities, there was improved access to effective malaria treatment in Ghana [19, 20]. Despite these laudable interventions, there are still challenges experienced in malaria control in Ghana [18]. ITNs ownership coverage is uneven within the country with 42% in greater Accra and 52% in the Volta Region [20]. More so, ownership does not translate to usage, as only 42% of those who own ITN sleep under it [20]. Besides, improvement in the healthcare system has not improved the malaria treatment-seeking behaviour of the people, as reports show that some people still use self-medication, herbal treatment and other informal treatment in the management of malaria [21, 22]. With the changing and uneven trends of malaria prevention activities in the country, it is very apt for a constant evaluation of the progress of malaria control and prevention programmes at the district and regional levels in Ghana. Thus, this study was conducted to assess health-seeking behaviour and malaria prevention practices in four districts in the Volta Region of Ghana.

## Methods

### Study site and population

The survey was conducted in the Volta Region of Ghana. The region is one of the 16 regions in the country (previously 10) (Ghana Statistical Service [GSS], 2013). The Volta Region is located between latitudes 50° 45″N and 80° 45″N along the southern half of the eastern border of Ghana, which it shares with the Republic of Togo. The region shares boundaries to the west with Greater Accra, Eastern and Brong Ahafo regions, to the north with the Northern Region, and has the Gulf of Guinea to the south. The region’s total land area is 20,570 square kilometres, representing 8.7% of the total land area of Ghana (GSS, 2013); and a population of 1,865,332, with about 72% living in rural areas. The Volta Region has a total of 326 health institutions, out of which 242 are administered by the GHS, 18 are missionary-owned, one is quasi-government, and 65 are privately owned (GSS, 2013).

### District characteristics

The study was conducted in four districts/municipalities out of the 18 districts/municipalities of the Volta Region. These four districts/municipalities include Ho West District, Ketu South, Nkwanta South (now in the newly created Oti Region) and Hohoe Municipalities. According to the 2010 Population and Health Census of Ghana, the total number of households in Hohoe is 43,329, Ketu South is 39,119, while Ho West and Nkwanta South have 23,875 and 22,733 respectively. Ketu south municipality is one of the study sites with the highest percentage of urban population (46%), which according to the Ghana statistical service population health census data of 2010 makes it the second most urbanized municipality in Ghana, second to Keta municipality [23]. Nkwanta south municipality has the highest percentage of people who are defined as illiterates [24], while the percentage of population employed for all the four districts in this study is almost similar [25, 26]**.** The study population from these sites comprised men and women aged 18–70 years from selected households (Table 1).

**Table 1:** District characteristics

Characteristics of districts	Ho west	Ketu south	Nkwanta south	Hohoe
Population	94,600	160,756	117,878	167,016
Total # House hold	23,875	39,119	22,733	43,329
%population in urban areas	10.9%	46%	25.6%	52.6%
population of > 15	60,069	100,044	64,832	107, 085
% population of illiterate	14.1%	28.1%	47.3%	11.7%
% population of employed	62.4%	64.5%	68.3	60.5

SOURCE: Districts analytical report, GSS (2014)

### Study design

This was a cross-sectional household survey involving a multi-staged sampling of household members between the ages of 18 to 70 years. The first stage involved random sampling of four districts from the three ecological zones (savanna, middle, and coastal zones) of the region. Ketu South district was selected for the coastal ecological zone, Nkwanta South district for the savanna, while Ho West and Hohoe were selected for the middle zone.

The second stage of the sampling involved the use of a simple random sampling of thirty communities from each of the study sites; and in each community, fifteen households were randomly selected for the third stage. For the selection of the 15, the random walk technique was adopted. With this technique, interviewers selected the first housing unit by moving clockwise from the centre of the community and then followed a specific path of travel to select the rest of the housing units. Finally, the fourth stage of the sampling involves the random sampling of the available adults in each household sampled.

### Sample size

Based on the 2010 populations of the household for the four districts’ populations, Yamane’s (1998) formula for sample size determination was used to determine the minimum number of participants for this study.

With the formula n = $\frac{N}{1 + N( \propto)^{2}}.$ Where n is the minimum sample size to be determined, N is the study population, and α is the margin of error which was 0.05 at a significance level of 95% and adding 10% non-response rate, the minimum participants for the four districts were 436 for Ketu South, 433 for Ho West, 437 for Hohoe, and 432 for Nkwanta South resulting in a minimum sample size total of 1738 participants.

### Data collection procedure

Data were collected with a structured questionnaire electronically administered using a Computer Assisted Personal Interview (CAPI) installed in smartphones of data collectors. The data collection exercise took place from February to April 2019 from 9 am to 2 pm each weekday. Information to highlight the sociodemographic characteristics of the participants were obtained, and these included age, gender, level of education, religion and occupation.

Occupation was categorized into farming, trading, skilled and unskilled labour, civil servants, unemployed and “others”. Farming includes those involved in animal rearing and land cultivation, while trading includes both petty and large-scale trading. Skilled labour includes electricians, painters, carpenters and other skilled artisans, while unskilled labour includes porters, cleaners, bus conductors. Civil servants are all those employed in the formal sector, including teachers, accountants, healthcare workers, security personnel, administrators. Unemployed includes those without any form of employment and source of income, and these include students and other adults without employments. Other category of occupation, designated as “others”, includes volunteer workers, town criers, priests, pastors and imams, toll collectors.

Questions on health-seeking behaviour were also asked; they included: whether fever was experienced in the past 3 days, and what was done after the onset of symptoms. Questions on ownership, use of ITN and the use of sprays and/or coils, as well as the use of other vector control practices, were also asked.

### Data analysis

Electronic data were downloaded into a single master (Microsoft excel 2016 spreadsheet) database following completion of the fieldwork and Statistical Package for the Social Sciences (SPSS) version 22, was used for analysis. Descriptive statistics comprising frequencies and proportions were used to summarize sociodemographic variables and malaria prevention practices among the participants. Chi-square was used to test for significance of associations between socio-demographic variables and malaria prevention practices such as ownership and use of ITN, and health-seeking behaviour like actions taking after the onset of fever among participants. Multivariate nominal logistic regression analysis was used to model the relationship between dichotomous dependent variables and independent variables. The dichotomous dependent variables are ITN ownership and ITN usage. The independent variables used in the multivariate logistic regression were sociodemographic variables that had significant association with ITN Usage and Ownership in Chi-square analysis. Odds ratios, 95% confidence limits and p-values were reported. All analyses were done using 95% confident intervals and statistical significance set as p ≤ 0.05.

### Ethical considerations

Ethical clearance for the survey was obtained from the UHAS Review Ethics Committee (UHAS-REC A.6 [7] 17/18). Permission was also sought from the district/municipal health directorates and traditional authorities of the various communities before data were collected. Written informed consent was obtained from participants before including them in the study. Confidentiality was also ensured by using initials and questionnaire number codes instead of real names.

## Results

### Socio-demographic characteristics

The sociodemographic characteristics are summarized in Table 2. A total of 2493 participants took part in this study, with 56% (1400) and 44% (1093) comprising females and males, respectively. Most participants were between the ages of 18–29 (612; 25%), while the least number of them is seen among those from 70 years and above (151; 6.1%). Ketu south district had the highest number of participants (834; 34%), while Nkwanta south had the least number of participants (446; 18%). Christianity was the modal religion among the participants (2154; 86%); and the majority had Junior high school (JHS) as their highest level of education (994; 40%). Farming was the highest occupation of the participants (742; 30%), while 283 (11%) of them were unemployed (Table 2).

**Table 2:** Socio-demographic characteristics

Characteristics	Frequency	%
*Gender*		
Male	1093	43.8
Female	1400	56.2
*Age*		
18–29	612	24.5
30–39	605	24.3
40–49	509	20.4
50–59	392	15.7
60–69	224	9.0
≥ 70	151	6.1
*Districts*		
Ketu South	834	33.5
Ho West	671	26.9
Hohoe	542	21.7
Nkwanta South	446	17.9
*Religion*		
Christianity	2154	86.4
Islam	96	3.9
Traditional	243	9.7
*Education*		
None	357	14.3
Primary	372	14.9
JHS	994	40.0
SHS	532	21.3
Tertiary	238	9.5
*Occupation*		
Farming	742	29.8
Trading	685	27.5
Skilled labour	437	17.5
Unskilled labour	139	5.6
Civil servants	155	6.2
Unemployed	281	11.2
Others	54	2.2

### Health-seeking behaviour and malaria prevention practices

Table 3 shows health-seeking behaviour and malaria prevention practices among the participants. A total of 668 (27%) participants affirmed that they had experienced fever within the past 3 days of the interview, of which 156 (23%) reported they visited the hospital after the onset of the fever, 268 (40%) patronized the pharmacy, 62 (9.3%) had recourse to herbal treatment, and 140 (21%) reported they did not do anything. Nine (9) in 10 participants in this study stated they owned at least one ITN (2234; 89.6%) which was predominantly obtained for free from health facilities (1367; 61%). Of those who owned ITN, 1528 (68%) stated they used them the night before the study. Seven hundred and sixty-eight (768; 31%) and 368 (15%) of the participants reported using mosquito sprays and mosquito coils, respectively. Table 3 also illustrates other actions taking by the 42 participants after their onset of fever. The majority of them took alcoholic bitters (18; 42.9%) and (17; 40.5%) took leftover drugs at home.

**Table 3:** Health-seeking behaviour and malaria prevention practices

Characteristics	Frequency	%
*History of Fever within 3 days*		
Yes	668	26.8
No	1825	73.2
*Actions taken during fever*		
Visited a health facility	156	23.4
Visited a patent medicine store/pharmacy	268	40.1
Visited the herbalist	62	9.3
Did not do anything	140	20.9
Others	42	6.3
*Ownership of ITN*		
Owned ITN	2234	89.6
Did not own ITN	259	10.4
*Source of ITN*		
Bought personally	51	2.3
from community programmes	793	35.5
from a health facility	1367	61.2
Gift from family member/friend	23	1.0
*Use of ITN previous night (n = 2234)*		
Used	1528	68.4
Did not use	706	31.6
*Use of mosquito spray*		
Yes	768	30.8
No	1725	69.2
*Use of mosquito coil*		
Yes	368	14.8
No	2125	85.2
Use neither ITN, mosquito spay or coil	413	16.6
*Other actions taken during fever*		
Took left over drugs	**17**	**40.5**
Took a lot of water	**6**	**14.2**
Took alcoholic bitters	**18**	**42.9**
Prayed	**1**	**2.4**

Other vector control technique employed by participants.

**Fig. 1:**
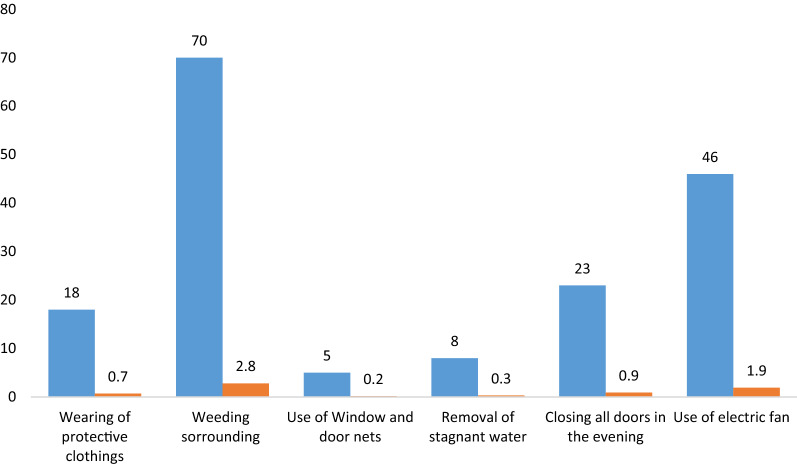
Other vector control technique employed by participants

Out of 2493 participants in this study, 70 (2.8%) participants indicated they weed the surrounding areas around their houses as a means to reduce the number mosquitoes, 46 (1.9%) stated they use electric fans, 23 (0.9%) said they close all doors and windows in the evening, 18 (0.7%) mentioned wearing protective clothing, and 8 (0.3%) stated removing and draining stagnant waters around their homes (Fig. 1).

### Characteristics of participants stratified by actions taken after the onset of fever

Table 4 shows the association between the sociodemographic variables and the actions taken after the onset of fever by the 668 participants who had a fever within the past 3 days. There was no significant difference seen in the actions taken after the onset of fever concerning sex, district, religion and highest educational level (p > 0.05) However, there was a significant difference noted among the different occupations (p = 0.001), with civil servants (7, 30.4%) and traders (57; 30.5%) constituting the highest proportions of participants who visited the hospital after the onset of fever and the lowest proportion seen among the unemployed (14; 17.3%). Furthermore, civil servants (11; 47.8%) were among the highest proportion of occupation that patronized patent medicine store only slightly below skilled labourers which constituted the highest proportion (67; 52.3%). Unskilled labourers constituted the highest proportion of participants that resorted to herbal treatment (6; 17.6%) followed by traders (27; 14.4%). The unemployed 30 (37%) constituted the highest proportion of participants that resorted to doing nothing after the onset of fever followed by unskilled labourers 9 (26.5%).

**Table 4:** Characteristics of participants stratified by actions taken after the onset of fever

Characteristics	Health facility N (%)	Patent medicine store N (%)	Herbal treatment N (%)	Nothing N (%)	Others N (%)	Total	P
*Gender*							
Male	45 [17.7]	113 [44.5]	28 [11.0]	51 [20.1]	17 [6.7]	254 [38]	0.06
Female	111 [26.8]	155 [37.4]	34 [8.2]	89 [21.5]	25 [6.0]	414 [62]	
*Age*							
18–29	39 [25.3]	64 [41.6]	9 [5.8]	37 [24.0]	5 [3.2]	154 [23.1]	
30–39	35 [19.6]	80 [44.7]	16 [8.9]	35 [19.6]	13 [7.3]	179 [26.8]	
40–49	28 [19.2]	65 [44.5]	17 [11.7]	24 [16.4]	12 [8.2]	146 [21.9]	
50–59	24 [24]	30 [30	12 [12	27 [27]	7 [7]	100 [14.9]	0.13
60–69	18 [35.3]	15 [29.4]	6 [11.8]	9 [17.6]	3 [5.9]	51 [7.6]	
≥ 70	12 [31.6]	14 [36.8]	2 [5.3]	8 [21.1]	2 [5.3]	38 [5.7]	
*Districts*							
Ketu South	56 [23.1]	96 [39.7]	26 [10.7]	50 [20.7]	14 [5.8]	242 [36.2]	
Ho West	35 [24.6]	48 [33.8]	15 [10.7]	34 [23.9]	10 [7.0]	142 [21.3]	0.3
Hohoe	31 [21.9]	66 [46.8]	7 [4.9]	32 [22.8]	5 [3.6]	141 [21.1]	
Nkwanta South	34 [23.8]	58 [40.6]	14 [9.8]	24 [16.8]	13 [9.1]	143 [21.4]	
*Religion*							
Christianity	137 [24.8]	222 [40.2]	44 [7.9]	120 [21.7]	29 [5.3]	552 [82.6]	
Islam	5 [19.2]	7 [26.9]	4 [15.4]	6 [23.1]	4 [15.4]	26 [3.9]	0.1
Traditional	14 [15.6]	39 [43.3]	14 [15.6]	14 [15.6]	9 [10]	90 [13.5]	
*Education*							
None	25 [22.5]	51 [45.9]	11 [9.9]	16 [14.4]	8 [7.3]	111 [16.6]	
Primary	25 [20.5]	48 [39.3]	14 [11.5]	25 [20.5]	10 [8.2]	122 [18.3]	0.4
JHS	61 [21.9]	107 [38.4]	24 [8.6]	71 [25.4]	16 [5.7]	279 [41.8]	
SHS	34 [28.3	46 [38.3]	11 [9.2]	23 [19.2]	6 [5.0]	120 [17.9]	
Tertiary	11 [30.6]	16 [44.4]	2 [5.6]	5 [13.9]	2 [5.6]	36 [5.4]	
*Occupation*							
Farming	46 [22.9]	81 [40.3]	18 [8.9]	39 [19.4]	17 [8.5]	201 [30.1]	
Trading	57 [30.5]	63 [33.7]	27 [14.4]	30 [16.0]	10 [5.3]	187 [27.9]	
Skilled labour	23 [17.9]	67 [52.3]	7 [5.5]	27 [21.1]	4 [3.1]	128 [19.2]	0.001
Unskilled labour	6 [17.6]	10 [29.4]	6 [17.6]	9 [26.5]	3 [8.8]	34 [5.1]	
Civil servants	7 [30.4]	11 [47.8]	1 [4.3]	2 [8.7]	2 [8.7]	23 [3.4]	
Unemployed	14 [17.3]	31 [38.3]	2 [2.5]	30 [37.0]	4 [4.9]	81 [12.1]	
Others	3 [21.4]	5 [35.7]	1 [7.1]	3 [21.4]	2 [14.4]	14 [2.1]	

### Characteristics of participants stratified by the ownership and usage of ITN

Table 5 shows the association between sociodemographic variables and ITN ownership and usage. ITN ownership was significantly seen more with females (1293: 92.4%, p ≤ 0.001). Though females (903, 69.8%) slept more under ITN compared to males (625 66.4%), this finding was not significant (p = 0.13). There was no significant association between ITN ownership and the age groups (p = 0.16). However, the participants in the above 70 years age group constituted the significant lowest proportion of ITN non-usage (37; 26.8%) and the highest proportion of ITN usage (101; 73.2%)(p = 0.04). All four districts have over 85% ownership of ITN with Nkwanta South Municipality significantly having the highest proportion (424; 95.1%, p ≤ 0.001) and also had the significant highest proportion of participants who used ITN (355; 83.7%, p ≤ 0.001). Religion did not have a significant association (p = 0.1) with ownership of ITN even though participants who are traditionalist had the highest proportion (225, 92.6%). The usage of ITN was however significant in its association with religion with participants who are traditionalist having the highest proportion (182; 80.9%, p ≤ 0.001) of usage. Education had a significant association with ITN ownership (p = 0.003) and usage (p ≤ 0.001). Participants who are farmers constituted the highest proportion of those who owned (688; 92.7%, p ≤ 0.001) and used ITN (531; 77.2%, p ≤ 0.001).

**Table 5:** Characteristics of participants stratified by the ownership and usage of ITN

Characteristics	Ownership of ITN		P-value	Usage of ITN		P-value
	Yes	No		Yes	No	
*Gender*						
Male	941 [86.1]	152 [13.9]	≤ 0.001	625 [66.4]	316 [33.6]	0.13
Female	1293 [92.4]	107 [7.6]		903 [69.8]	390 [30.2]	
*Age*						
18–29	533 [87.1]	79 [12.9]	0.16	358 [67.2]	175 [32.8]	0.04
30–39	546 [90.2]	59 [ [9.8]		390 [71.4]	156 [28.6]	
40–49	464 [91.2]	45 [8.8]		294 [63.4]	170 [36.6]	
50–59	355 [90.6]	37 [9.4]		247 [69.6]	108 [30.4]	
60–69	198 [88.4]	26 [11.6]		138 [69.7]	60 [30.3]	
≥ 70	138 [91.4]	13 [8.6]		101 [73.2]	37 [26.8]	
*Districts*						
Ketu South	720 [86.3]	114 [13.7]	≤ 0.001	477 [66.3]	243 [33.7]	≤ 0.001
Ho West	608 [90.6]	63 [9.4]		398 [65.5]	210 [34.5]	
Hohoe	482 [88.9]	60 [11.1]		298 [61.8]	184 [38.2]	
Nkwanta South	424 [95.1]	22 [4.9]		355 [83.7]	69 [16.3]	
*Religion*						
Christianity	1925 [89.4]	229 [10.6]	0.1	1280 [66.5]	645 [35.5]	≤ 0.001
Islam	84 [87.5]	12 [12.5]		66 [78.6]	18 [21.4]	
Traditional	225 [92.6]	18 [7.4]		182 [80.9]	43 [19.1]	
*Education*						
None	329 [92.2]	28 [7.8]	0.003	265 [80.5]	64 [19.4]	≤ 0.001
Primary	329 [88.4]	43 [11.6]		244 [74.2]	85 [25.8]	
JHS	907 [91.2]	87 [8.8]		609 [67.1]	298 [32.9]	
SHS	460 [86.5]	72 [13.5]		290 [63.0]	170 [37.0]	
Tertiary	209 [87.8]	29 [12.2]		120 [57.4]	89 [42.6]	
*Occupation*						
Farming	688 [92.7]	54 [7.3]	≤ 0.001	531 [77.2]	157 [22.8]	≤ 0.001
Trading	620 [90.5]	65 [9.5]		398 [64.2]	222 [35.8]	
Skilled labour	383 [87.6]	54 [12.4]		244 [63.7]	139 [36.3]	
Unskilled labour	115 [82.7]	24 [17.3]		82 [71.3]	33 [28.7]	
Civil servants	134 [86.5]	21 [13.5]		85 [63.4]	49 [36.6]	
Unemployed	244 [86.8]	37 [13.2]		154 [63.1]	90 [36.9]	
Others	50 [92.6]	4 [7.4		34 [68]	16 [32.0]	

### Multivariate logistic regression analysis of the odds of owning and using an ITN

Tables 6 and 7 show the multivariate logistic regression analysis of the odds of owning and using ITN among participants respectively. Participants from Ketu South significantly had higher odds of owning a net (Unadjusted OR, 1.60 [95% 1.14–2.24]; p = 0.01]; Adjusted OR, 1.56 [95% 1.09–2.22]; p = 0.01) (Table 6). Participants from Nkwanta South were significantly less likely to own an ITN (Unadjusted OR, 0.51 [95% 0.35–0.85]; p = 0.01]; Adjusted OR, 0.49 [95% 0.29–0.86]; p = 0.01) (Table 6). Also, participants from Nkwanta South were less likely to use ITN (Unadjusted OR, 0.35 [95% 0.25–0.48]; p ≤ 0.001]; Adjusted OR, 0.40 [95% 0.29–0.57]; p ≤ 0.001) (Table 7). Participants with no education (Adjusted OR, 0.32 [95% 0.20–0.50]; p ≤ 0.001), primary (Adjusted OR, 0.42 [95% 0.28–0.64]; p ≤ 0.001) and JHS (Adjusted OR, 0.61 [95% 0.43–0.87]; p ≤ 0.001) had significantly lower odds of using ITN, respectively (Table 7).

**Table 6:** Multivariate logistic regression analysis of the odds of owning ITN

Characteristics	Unadjusted OR (95% CI)		Adjusted OR (95% CI)	
*Gender*				
Female	0.48 (0.36–0.610	0.10	0.55 (0.39–0.69)	0.12
Male		1	1	
*Districts*				
Ketu South	1.60 (1.14–2.24)	0.01	1.56 (1.09–2.22)	0.01
Nkwanta South	0.51 (0.30–0.85)	0.01	0.49 (0.29–0.86)	0.01
Hohoe	1.29 (0.88–1.89)	0.19	1.23 (0.83–1.81)	0.31
Ho West	1		1	
*Occupation*				
Farming	0.65 (0.22–1.89)	0.43	0.66 (0.22–2.04)	0.47
Trading	0.96 (0.33–2.78)	0.91	1.37 (0.46–4.06)	0.58
Skilled labour	1.39 (0.48–4.04)	0.55	1.34 (0.45–0.395)	0.6
Unskilled labour	2.25 (0.73–6.90)	0.16	1.87 (0.59–5.85)	0.25
Civil servants	1.62 (0.54–5.14)	0.38	1.46 (0.46–4.99)	0.52
Unemployed	1.47 (0.49–4.35)	0.40	1.65 (0.54–4.99)	0.37
Others	1		1	
*Education*				
None	0.55 (0.31–0.98)	0.04	0.89 (0.47–1.73)	0.75
Primary	0.85 (0.50–1.44)	0.55	1.27 (0.70.2.30)	0.43
JHS	0.64 (0.40–1.02)	0.06	0.95 (0.57–1.59)	0.86
SHS	1.15 (0.72–1.85)	0.56	1.50 (0.89–2.55)	0.13
Tertiary	1		1	

*OR* odds ratio, *CI* confidence interval

**Table 7:** Multivariate logistic regression analysis of the odds of using ITN

Characteristics	Unadjusted OR (95% CI)		Adjusted OR (95% CI)	
*Districts*				
Ketu South	0.93 (0.74–1.18)	0.50	1.04 (0.81–1.33)	0.73
Nkwanta South	0.35 (0.25–0.48)	≤ 0.001	0.40 (0.29–0.57)	≤ 0.001
Hohoe	1.18 (0.92–1.52)	0.20	1.20 (0.93–1.56)	0.162
Ho West	1		1	
*Occupation*				
Farming	0.65 (0.22–1.89)	0.008	0.56 (0.27–1.12	0.12
Trading	0.96 (0.33–2.78)	0.59	1.24 (0.61–2.51)	0.56
Skilled labour	1.39 (0.48–4.04)	0.67	1.11 (0.54–2.27)	0.78
Unskilled labour	2.25 (0.73–6.90)	0.31	0.80 (0.30–1.50)	0.59
Civil servants	1.62 (0.54–5.14)	0.90	0.76 (0.35–1.66)	0.49
Unemployed	1.47 (0.49–4.35)	0.82	1.19 (0.37–2.47)	0.65
Others	1		1	
*Education*				
None	0.29 (0.19–0.43)	≤ 0.001	0.32 (0.20–0.50)	≤ 0.001
Primary	0.40 (0.28–0.54)	≤ 0.001	0.42 (0.28–0.64)	≤ 0.001
JHS	0.57 (0.42–0.78)	0.001	0.61 (0.43–0.87)	0.006
SHS	0.73 (0.22–1.02)	0.68	0.73 (0.57–1.07)	0.11
Tertiary	1		1	
*Religion*				
Christianity	2.19 (1.51–3.18)	≤ 0.001	1.77 (1.17–2.67)	0.01
Islam	1.98 (1.05–3.75)	0.04	1.84 (0.93–3.64)	0.08
Traditional	1		1	

*OR* odds ratio, *CI*  confidence interval

## Discussion

Fever was a fairly common reported complaint among participants (668) in this study; and it is usually a common malaria symptom that triggers the decision to seek care compared to other symptoms [10]. Fever from malaria is not easily differentiated from other causes of febrile illnesses that are replete in malaria endemic areas, and that was why the WHO recommend prompt malaria testing in patients presenting with fever [27]. Prompt malaria testing is an important component of effective case management control strategy aimed at preventing malaria mortality [8, 10, 27]. The success of this control strategy vis-à-vis prompt malaria detection and treatment among persons with fever is dependent of health-seeking behaviour of those involved [8–10].

More than half of the 668 participants who admitted to having had fever within the past 3 days, reported to have either sought help from a patent medicine store (40%) or visited a health facility (23%). The high patronage of patent medicine stores or vendors is not surprising as they are replete in many African rural settings where they provide care for patients, especially prompt malaria treatment [28–30]. Though they are not usually staffed and overseen by trained pharmacists, and not stocked with expensive malaria drugs, they are often stocked with basic and effective artemisinin-based combination therapy (ACT) dispensed by a trained medicine counter assistant [30]. More so, they are often equipped with simple RDTs to quickly arrive at a malaria diagnosis in patients with fever [29, 31]. Visiting health facilities after the onset of fever was the second-best option for participants in this study; and it was significantly reported among civil servants and traders, probably because of the financial implications vis-a-vis the relatively higher out of pocket expenses compared to patent medicine stores [28, 32, 33]. Out of pocket expenses influence treatment-seeking behaviour for malaria, as only those with adequate finances can have the satisfaction of spending more to get the best care when sick with malaria [34]. Like most Low-and-Middle-Income-Countries (LMIC), Ghana’s National Health Insurance Scheme (NHIS) is plagued with poor coverage of about 40% of the population and several other challenges, making the attainment of Universal Health Coverage (UHC) a big challenge in the country [35, 36]. This has made many in Ghana spend so much in out of pocket expenses in seeking health care, especially for recurrent diseases like malaria [35]; thus making the poor and those without adequate finances seek cheaper alternative treatments, such as herbal remedies [34]. Only 62 participants agreed to having taken herbal treatment for their fever, which is a rather common practice in malaria-endemic areas including Ghana [37, 38]. Another group of participants claimed not to have done anything since the onset of their fever within the past three days, probably still observing the symptoms to either take action if symptoms persist or worsen [39]. This attitude usually leads to delay in treatment resulting in the complication of malaria especially in vulnerable groups such as children and pregnant women [30].

ITN ownership and usage (sleeping under ITN) among the participants in this study was 90% and 68%, respectively. This trend of low usage of ITN despite relatively high ownership has been reported in many parts of the country [20, 40–43]. There are several reasons people give for not sleeping under ITN despite having one at home. These include the inability to mount the ITN due to lack of appropriate location in the household, complaints that sleeping under the ITN is uncomfortable due to increase warmth (due to them feeling warmer under the net), and not enough ITN to accommodate every member of the household [44, 45]. Aside these complaints, there is however inappropriate use of the ITNs, as it has been reported that some people use them for fishing, farming and other activities [46, 47]. The majority of the ITNs in this study were obtained through free means, either through health facilities (61%) or community outreach programmes (36%); and this might contribute to the reason for the inappropriate usage of the ITNs [45, 48].

Females (92%) were found to be significantly (p ≤ 0.001) more likely to own ITNs than males (86%) in this study; and, although more women slept under the ITN, this finding was not statistically significant. This finding has been reported in some other studies [49, 50]. The likelihood of females especially those of reproductive age to visit health facilities when pregnant or participate in community outreach programmes where free ITNs are distributed might explain this finding [51, 52]. Females are also prioritized to sleep under the ITNs when there are not enough ITNs in the household, and they often share ITNs with younger children [49, 50]. Participants from Ketu south were 1.5 times more likely to own an ITN, while participants from Nkwanta south were less likely to sleep under an ITN. This finding might have been influenced by the varied sociodemographic characteristics of the districts [23, 24]. The high % population of people living in urban areas in Ketu south municipality, as well as the relative high number of people that are literate, and the opposite seen in Nkwanta south might have contributed to these findings. Education seems to influence the use of ITNs in this study; a finding similarly reported in some other studies, as participants with lower levels of education were less likely to use ITN [53–55].

Mosquito spray and coil were other methods of vector control used by participants in this study. Mosquito coils are usually cheaper alternatives compared to mosquito aerosol spray for those who do not have ITN, and it is widely used in several malaria-endemic countries including Ghana [16, 43, 55]. Mosquito aerosol spray is also widely used in malaria-endemic areas including Ghana [17, 43, 56–58]. There are no official approval or recommendation by the National Malaria Control Programme or the Ghana Health Service on the use of mosquito aerosol sprays and coils, but their use seem to be common in Ghana as some prefer them to ITNs because of easy availability and applicability [43]. Although this study observed ITN use together with mosquito spray and coil, it is very likely that in reality they were not used together as the use of mosquito spray and coil often reduced the likelihood of the use of ITN [43]. The findings in this study show that only a few of the participants relied on mosquito spray and/or mosquito coil, a finding similar to studies done in Sunyani in Ghana and Imo State in Nigeria [43, 59].

This study had better ITN ownership and usage compared to some studies in other parts of Ghana [20, 41–43]. This relatively higher ITN ownership and usage might have been influenced by the rainy season when this study was done, which normally increase ITN ownership and usage [43, 60]; though this influence was not seen in ITN ownership and usage in the study done in Sunyani in Ghana [43]. However, a worrying finding is the 16.6% of the study participants who did not employ any form of vector control methods like ITN, mosquito coil and/or spray, which is much higher than 13.8% in the Sunyani study [43].

This study highlighted interesting findings concerning health seeking behaviour and malaria prevention practices in the Volta Region. However, the inherent complexities of health seeking behaviors and malaria preventive practices among people in communities are usually hard to conceptualize solely from responses of cross-sectional studies like this one. It might also be necessary that qualitative studies be employed to further explore the reasons behind the findings.

## Conclusion

The study showed that the majority of the participants who had a fever within the past 3 days visited patent medicine stores (40%) and health facilities (23%). ITN ownership was 90% and usage was 68% with more females reporting to own and use ITNs than males. Participants from Ketu South were 1.5 times likely to own an ITN, while those from Nkwanta South were less likely to use ITN. Mosquito aerosol sprays and coils were also used by few participants as vector control methods. Though the percentage of ITN ownership and usage among participants in this study were relatively higher than most studies done in Ghana, it is still below the national malaria control programme target; and more worrying is that about 17% of participants practice no form of vector control, and those using mosquito coils with proven health hazards. It is, therefore, very important that education on health-seeking behaviour vis-a-vis the importance of seeking help promptly and from the right place when ill with symptoms of malaria. Education should focus on the importance of going for malaria test when there is any febrile illness, to rule out other causes of fever. Education on malaria prevention practices like the proper use of ITNs and others should be intensified and continuous among the population of the Volta Region to ensure the success of malaria control in the region. Employing the appropriate behaviour change model might be needed to design an effective education programme.

## Data Availability

The datasets used during the current study are available from the corresponding author on reasonable request.
